# Roles of Plant-Derived Secondary Metabolites during Interactions with Pathogenic and Beneficial Microbes under Conditions of Environmental Stress

**DOI:** 10.3390/microorganisms7090362

**Published:** 2019-09-18

**Authors:** Kei Hiruma

**Affiliations:** 1 Department of Science and Technology, Nara Institute of Science and Technology, Nara 630-0192, Japan; 2 PRESTO, Japan Science and Technology Agency, 4-1-8 Honcho Kawaguchi, Saitama 332-0012, Japan; hiruma@bs.naist.jp

**Keywords:** *Arabidopsis thaliana*, *Colletotrichum*, indole glucosinolates, coumarins, nutrient deficiencies

## Abstract

Under natural conditions, plants generate a vast array of secondary metabolites. Several of these accumulate at widely varying levels in the same plant species and are reportedly critical for plant adaptation to abiotic and/or biotic stresses. Some secondary metabolite pathways are required for beneficial interactions with bacterial and fungal microbes and are also regulated by host nutrient availability so that beneficial interactions are enforced. These observations suggest an interplay between host nutrient pathways and the regulation of secondary metabolites that establish beneficial interactions with microbes. In this review, I introduce the roles of tryptophan-derived and phenylpropanoid secondary-metabolite pathways during plant interactions with pathogenic and beneficial microbes and describe how these pathways are regulated by nutrient availability.

## 1. Introduction

Plants face various abiotic and biotic stresses in nature. Unlike animals that can escape from these stresses, plants have to adapt to various types of stresses at the same time, and the evolution of a diverse repertoire of plant secondary metabolites is believed to be a central mechanism of adaptation [1]. It is now well documented that secondary metabolites play important roles in plant adaptation to biotic and abiotic stresses. Moreover, biosynthetic pathways for secondary metabolites that are involved in the adaptation to local environments are likely developed with specificity. For example, a recently described class of phenylacylated flavonols (saiginols) has been shown to be important for plant adaptation to high UV-B irradiation, especially in northern latitudes [2]. Tryptophan-derived indole glucosinolate biosynthetic pathways have specifically evolved in most Brassicaceae species, have wide inter- and intra-species variations in levels of accumulation, and are crucial for plant interactions with pathogenic and beneficial microbes [3,4,5,6,7]. Similar specific biological innovations of particular secondary metabolites are important for plant adaptations in specific plant lineages and have been frequently described in various plant species [8,9], further suggesting critical roles of secondary metabolites in plant adaptation to specific local environments.

Biosynthesis and/or secretion of secondary metabolites that are required for biotic interactions are often influenced by abiotic stresses, such as nutrient deficiencies. Hence, nutrient deficiencies have been central to the establishment of beneficial relationships between most land plants and microbes, and these ensure efficient nutrient uptake through symbiotic relationships [10]. Yet, plants still need to defend themselves against potential pathogens and must discriminate pathogenic from beneficial/commensal microbes. Although the systems by which such discrimination is achieved have not been described, recent reports suggest that plants meet these contrasting demands at least partly by regulating secondary metabolite pathways [11,12]. In this review, I discuss how plants regulate the biosynthetic pathways of secondary metabolites to achieve local adaptation and focus on plant interactions with beneficial microbes and microbiome interactions under nutrient-limiting conditions.

## 2. Tryptophan-Derived Secondary Metabolites Are Critical for Plant Interactions with Several Pathogenic and Beneficial Fungi

**Figure 1:**
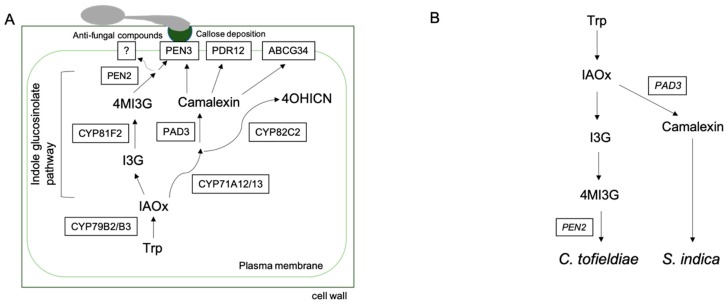
Overview of the tryptophan-derived metabolite pathway required for plant–microbe interactions. (**A**) Tryptophan (Trp) is converted to indole-3-acet aldoxime (IAOx) by the cytochrome P450 members CYP79B2 and CYP79B3. The indole glucosinolate pathway, the camalexin pathway, and the 4-hydroxyindole-3-carbonitrile (4OHICN) pathway are reportedly required for defense against pathogens. In the indole glucosinolate pathway, indol-3- ylmethyl glucosinolate (I3G) is converted to 4-methoxyindol-3-ylmethylglucosinolate (4MI3G) by the cytochrome P450 CYP81F2. 4MI3G is then hydrolyzed by the atypical myrosinase PEN2, and the resulting compounds are transported by the ABC transporter PEN3. In contrast, the weaker phenotype of *pen3* compared to *pen2* plants against non-adapted *Colletotrichum* fungi suggests that additional ABC transporters may participate in the export of metabolites generated by PEN2 [19]. In the camalexin pathway, both cytochrome P450 CYP71A12/CYP71A13 and PAD3 (CYP71B15) generate the phytoalexin camalexin. Camalexin is reportedly transported by PEN3, PDR12, and PDR6/ABCG34 [25,35]. CYP71A12/CYP71A13 and cytochrome P450 CYP82C2 are required for the generation of 4OHICN, which is required for defense against both fungal and bacterial pathogens. (**B**) The indole glucosinolate pathway is required for beneficial interactions with the root endophyte *Colletotrichum tofieldiae* (Ascomycete). The camalexin pathway is required for beneficial interactions with the root endophyte *Serendipita indica* (Basidiomycete).

Brassicaceae plant species generate diverse sets of secondary metabolites that contain sulfur and nitrogen in their structures, collectively known as glucosinolates [4]. On the basis of the types of amino acids in their structures, glucosinolates can be divided into three main groups. Aliphatic glucosinolates are generally derived from leucine, valine, methionine, isoleucine, and alanine, whereas indole and aromatic glucosinolates are derived from tryptophan and phenylalanine or tyrosine, respectively [13]. Tryptophan-derived indole glucosinolates are well described in the context of pathogenic and beneficial microbe interactions in leaves and roots. The functions of glucosinolates are dependent on the hydrolytic activities of specialized β-thioglucoside glucohydrolases, which are known as myrosinases and comprise a diverse subfamily of β-glucosidases [14]. Genetic analyses and determinations of secondary metabolite concentrations indicate that PEN2-mediated atypical myrosinase-dependent breakdown of the specific IG 4-methoxyindol-3-ylmethylglucosinolate (4MI3G) and subsequent GSH conjugation by GSTU13 are needed for broad-spectrum defense against invasion and growth of a diverse range of nonadapted and adapted pathogenic fungi [15,16,17,18,19,20,21,22] (Figure 1A). The generation of 4MI3G depends on a CYP81F2 P450 monooxygenase that is responsible for the hydroxylation of indol-3-ylmethyl glucosinolate (I3G; [16]). Although the compounds downstream of PEN2 that are responsible for the defense against pathogenic fungi have not yet been identified, genetic analyses suggest that the ABC transporter PEN3 is required for the secretion of several antimicrobial compounds, including PEN2-derived putative compounds, from apoplasts during the entry of fungal pathogens [23,24,25,26]. Myrosinases are considered peculiar to myrosin cells and are separated from other organelles. Moreover, only when insects or herbivores undermine cell integrity by damaging tissues, are they mixed with glucosinolate derivatives [27,28]. Under these conditions, myrosinases generate bioactive products, such as isothiocyanates [4]. However, the PEN2 atypical myrosinase initiates glucosinolate degradation without detectable tissue damage during pre-invasive defense responses against non-adapted pathogens [16]. This pathway is also involved in callose deposition, one of the defense-related outputs activated upon a microbe-associated molecular pattern (MAMP) treatment without any microbial entry attempt [29]. In addition, in a manner dependent on PEN2, SA-dependent cell death responses after treatments with MAMP were strongly observed in *nsl1* mutants in which the putative membrane attack complex/perforin (MACPF) domain was made defective [30]. These results suggest diverse roles of the indole glucosinolate pathway, likely including signaling roles. Yet, *pen2* plants were less susceptible to pathogenic fungi than *cyp79B2 cyp79B3* plants, which have defects in the first step of tryptophan-derived secondary metabolite biosynthesis, suggesting that other PEN2-independent metabolites contribute to defense responses [16,18,20,31] (Figure 1A). Partial roles of CYP71A12/CYP71A13-dependent branches, including the indolic phytoalexin camalexin biosynthetic pathway, the 4-OH-ICN pathway, and possibly the indole-3-carboxylic acid (ICA) pathway(s), have been reported during fungal growth in plant tissues [32,33,34] (Figure 1A).

The PEN2-dependent pathway is also required for plant interactions with beneficial fungal species. Indeed, plant growth promotion by the root endophyte *C. tofieldiae* (Ascomycete) under low Pi conditions is reportedly compromised in *pen2* mutant plants (Figure 1B, [7]). Compromised PGP in these *pen2* mutant plants was not, however, related to *C. tofieldiae* overgrowth in roots, whereas *C. tofieldiae* overgrowth was clearly observed in *cyp79B2 cyp79B3* mutant roots. These observations suggest that defects in *C. tofieldiae*-mediated PGP in *pen2* mutants is not due to overgrowth, but rather reflects potential roles of this pathway in beneficial interactions that do not restrict microbe growth. Further analyses are required to prove this idea. Interestingly, it has been reported that β-Lactam antibiotics strongly suppress the expression of *CYP81F2* that are upstream of *PEN2* (Figure 1A) and negatively influence the interaction with *C. tofieldiae* [36]. As the antibiotics can be predicted to be generated by bacteria in soil, the result suggests that the interplay between plants and soil- and/or root-associated bacteria influence the beneficial association via regulation of the indole glucosinolate pathway.

Importantly, even *C. tofieldiae* became pathogenic in *cyp79B2 cyp79B3* mutant plants under laboratory conditions. This life-style transition from beneficial to pathogenic in *cyp79B2 cyp79B3* mutant plants has been also reported for other distantly related beneficial fungi (Basidiomycetes), such as *Serendipita indica* (syn. *Piriformospora indica*) and *Sebacina vermifera* [5,6], indicating critical roles of tryptophan-derived metabolites, including indole glucosinolates, as moderators of symbiosis with fungi. In contrast with *C. tofieldiae*, however, the analysis of *cyp81F2* plants implies that indole glucosinolate pathways are not essential for the beneficial interactions with *S. indica* and *S. vermifera*, despite the requirement of the phytoalexin camalexin pathway for the beneficial interactions with *S. indica* [5]. Yet, camalexin appears not to be essential for beneficial interactions with *C. tofieldiae* [7] (Hiruma et al.; 2016). These genetic data suggest that the contributions of distinct branches of tryptophan-derived secondary metabolites are differentiated according to the life styles of fungal endophytes. At least, this seems to be the case for the camalexin pathway. Alternatively, in the case of indole glucosinolates, the responses of *cyp81F2* mutants to a necrotrophic fungal pathogen infection were weaker than those of *pen2* mutants [16]. Hence, redundancy of CYP81F family (CYP81F2 and CYP81F3) enzymes might hamper the assessment of their contributions during root colonization by specific types of fungi. In any case, it is important to identify critical compounds downstream of PEN2 for all of the responses described above.

## 3. Coumarins Shape Microbiome Composition in the Roots

Courmarins are plant secondary metabolites that are generated through the phenylpropanoid pathway [37]. Many roles of coumarins in abiotic and biotic stress responses have been shown [12] (Stringlis et al.; 2019). Several coumarins are excreted in root rhizospheres and are required for iron uptake under iron-deficient conditions [38]. Moreover, the related biosynthetic pathway is activated by the transcription factor MYB72 and by Feruloyl-CoA 6′-Hydroxylase1 (F6’H1) under iron-deficient conditions [39]. Iron is then sequestered by secreted coumarins that have Fe (III)-mobilizing activities, such as esculetin and flaxetin, and is absorbed by the iron-regulated transporter (IRT1) after reduction of Fe (III) to Fe (II) by the plasma membrane-resident ferric reduction oxidase 2 (FRO2; [39,40]). The basic helix-loop-helix protein FIT1 regulates most of the genes that are related to iron deficiency responses, including *MYB72*, *F6’H1*, and *IRT1*, and is required for growth in soil even with adequate nutrients [41]. Interestingly, MYB72 together with β-glucosidase BGLU42 regulated by MYB72 is also activated during beneficial bacterial root colonization under nutrient-deficient as well as -sufficient conditions and promotes induced systemic resistance in *A. thaliana* [42,43]. Although it is not currently clear whether this process is necessary for proper bacterial colonization in roots, these results indicate that root-associated bacteria modulate parts of the host nutrient signaling pathway, which provides host protection against several pathogens in systemic leaves.

In addition to the roles of coumarins in iron uptake, in vitro chemical assays showed that several coumarins, such as scopoletin, which is the most abundantly expressed coumarin in low-iron conditions, exhibit direct antimicrobial activities against several pathogens [12,44,45,46]. In any case, scopoletin from seed kernels of *Melia azedarach* shows synergistic anti-fungal effects when combined with other coumarins [47]. Considering that plants secret various types of coumarins, these results suggest that plants use a cocktail of metabolites to eliminate pathogenic microbes from the rhizosphere.

Recently, a coumarin biosynthetic pathway was shown to shape the bacterial microbiome in roots grown in soil and also to affect a synthetic bacterial microbiome comprising 22 phylogenetically diverse bacterial members from *A. thaliana* roots [12,48]. Comparative analysis of bacterial gene composition between WT and *f67h1* mutant plants revealed that scopoletin negatively influenced the abundance of certain bacterial groups, but positively influenced the amount of others [12]. Moreover, in vitro assays indicate that scopoletin inhibits the growth of some pathogenic fungi but not that of beneficial bacteria that induce biosynthesis and secretion of scopoletin. Furthermore, some microbial species use coumarins as a carbon source for growth [49,50,51]. Similarly, it has been recently reported that some of the root microbiota members convert anti-microbial triterpene compounds for their growth in vitro [9]. These data suggest that plants, possibly together with beneficial microbes, have developed a sophisticated system to eliminate only harmful pathogens from the rhizosphere by excreting coumarins that are useful for commensal and beneficial microbes and the microbiome. Further studies are required to reveal how the beneficial interactions have evolved and shaped the development of these systems.

## 4. Biosynthesis and/or Secretion Patterns of Secondary Metabolites That Are Related to Beneficial Interactions Depend on Nutrient Status

Glucosinolates have emerged as important factors in the interplay between nutrient deficiencies and plant–microbe interactions. Nutrients, such as phosphate, sulfate, and potassium, strongly influence glucosinolate biosynthesis [52,53,54]. For example, products of aliphatic glucosinolates are significantly influenced by phosphate availability (they are both up- and downregulated), which is mainly regulated by the master regulator of the plant phosphate starvation response (PSR) PHR1 [53]. Under conditions of potassium deficiency, several aliphatic and indole glucosinolates are accumulated, in part depending on the jasmonic acid signaling pathway [54]. In other examples, indole glucosinolates accumulate in broccoli plants grown under nitrogen-limiting conditions, whereas aliphatic glucosinolate levels are decreased under these conditions [55]. These reports suggest that glucosinolate quantities are not necessarily positively correlated with nutrient availability.

Importantly, nutrient-dependent control of glucosinolates in low-Pi conditions is linked to the establishment of beneficial interactions with root-associated fungi. During root colonization of beneficial *C. tofieldiae*, *A. thaliana* genes that are related to the biosynthesis or regulation of tryptophan-derived secondary metabolites are differentially regulated (mostly suppressed) under low-Pi conditions compared with sufficient-Pi conditions [11]. In contrast with the responses to beneficial *C. tofieldiae* under low-Pi conditions, plants strongly induce these genes and other defense-related genes during root colonization of pathogenic *C. incanum*, which is a close relative of *C. tofieldiae* [11]. These contrasting transcriptome patterns suggest that the attenuated expression of these genes during *C. tofieldiae* colonization is not simply due to limited phosphate availability. Rather, plants likely distinguish between pathogenic and beneficial fungi via unknown mechanisms and mediate their responses according to nutrient availability. Importantly, PHR1 and its paralog PHL1 restrict early root colonization of beneficial *C. tofieldiae* under low-Pi conditions [7]. Thus, PSR may regulate tryptophan-derived secondary pathways to accommodate beneficial microbes such as *C. tofieldiae*, which provides phosphate to its hosts. The interplay between PSR and plant–microbe interactions has also been demonstrated in a study of plant interactions with the root-associated bacterial microbiome [56,57]. How the production of these metabolites is influenced by host nutrient availability during root colonization by beneficial and pathogenic microbes, and by the microbiome, remains a subject of important future studies. To this end, it will be also necessary to identify compounds that are critical to these beneficial interactions.

Similar to glucosinolates, coumarin secretion in root exudates is significantly altered by low-iron and low-phosphate conditions [58,59]. Under low-Pi, coumarins, such as sculetin, flaxetin, and scopletin, are accumulated, whereas the other forms, especially the highly oxygenated forms, are suppressed. In contrast, low-iron conditions lead to increases in the levels of all types of coumarins [58,59]. Considering the roles of iron-dependent scopletin secretion in bacterial microbiome composition, this complex interplay between iron and phosphate likely influences plant interactions with microbes in the roots.

## 5. Conclusions and Future Perspectives

In this review, I introduce a few plant secondary metabolite pathways that are influenced by nutrient availability and have been identified as important factors for plant interactions with beneficial microbes and root-associated microbiomes. In addition to these metabolites, flavonoids are critical signaling molecules during the symbiosis of plants and nitrogen-fixing rhizobia, and the balance between carbon and nitrogen influences flavonoid biosynthetic pathways [60]. The production of the plant hormones strigolactones, which are required for attracting the mutualistic arbuscular mycorrhizal fungi, is also influenced by phosphate availability [61]. These observations strongly suggest that how plants sense and react to nutrient deficiencies in the context of plant secondary metabolites is key to the understanding of these beneficial interactions. Currently, the molecular mechanisms underlying plant responses to low-Pi, -N, and -Fe are described mainly in studies of model plants such as *Arabidopsis thaliana* and *Oryza sativa* [12,62,63,64,65,66]. As described above, many studies suggest relationships between nutrient-sensing pathways and plant secondary metabolites. However, with the exception of only a few cases, it is still not clear whether this interplay with microbes is direct or is a consequence of indirect responses that are generally related to nutrient deficiencies. It is also noteworthy that primary metabolites and other secondary metabolites influence the production of particular secondary metabolites [53,67]. Furthermore, because this interplay should be tightly related to direct and indirect nutrient transfer from beneficial microbes, it is necessary to address the microbial molecular mechanisms that underlie nutrient transfer. These remain poorly understood. Access to multiple genetic resources from model studies and the amenability of some microbe partners to molecular genetic manipulation will help to dissect the mechanisms of these important complex phenomena.
